# Electrolyte imbalances and dehydration play a key role in *Batrachochytrium salamandrivorans* chytridiomycosis

**DOI:** 10.3389/fvets.2022.1055153

**Published:** 2023-01-13

**Authors:** Wesley C. Sheley, Matthew J. Gray, Mark Q. Wilber, Carolyn Cray, E. Davis Carter, Debra L. Miller

**Affiliations:** ^1^Department of Biomedical and Diagnostic Sciences, College of Veterinary Medicine, University of Tennessee, Knoxville, Knoxville, TN, United States; ^2^Center for Wildlife Health, University of Tennessee Institute of Agriculture, Knoxville, TN, United States; ^3^Division of Comparative Pathology, University of Miami Miller School of Medicine, Miami, FL, United States

**Keywords:** amphibian, chytrid, clinical pathology, disease, histopathology, newt

## Abstract

**Introduction:**

One of the most important emerging infectious diseases of amphibians is caused by the fungal pathogen *Batrachochytrium salamandrivorans (Bsal)*. *Bsal* was recently discovered and is of global concern due to its potential to cause high mortality in amphibians, especially salamander species. To date, little has been reported on the pathophysiological effects of *Bsal*; however, studies of a similar fungus, *B. dendrobatidis (Bd)*, have shown that electrolyte losses and immunosuppression likely play a key role in morbidity and mortality associated with this disease. The goal of this study was to investigate pathophysiological effects and immune responses associated with *Bsal* chytridiomycosis using 49 rough-skinned newts (*Taricha granulosa*) as the model species.

**Methods:**

*Taricha granulosa* were exposed to a 1 × 10^7^ per 10 mL dose of *Bsal* zoospores and allowed to reach various stages of disease progression before being humanely euthanized. At the time of euthanasia, blood was collected for biochemical and hematological analyses as well as protein electrophoresis. Ten standardized body sections were histologically examined, and *Bsal*-induced skin lesions were counted and graded on a scale of 1–5 based on severity.

**Results:**

Results indicated that electrolyte imbalances and dehydration induced by damage to the epidermis likely play a major role in the pathogenesis of *Bsal* chytridiomycosis in this species. Additionally, *Bsal*-infected, clinically diseased *T. granulosa* exhibited a systemic inflammatory response identified through alterations in complete blood counts and protein electrophoretograms.

**Discussion:**

Overall, these results provide foundational information on the pathogenesis of this disease and highlight the differences and similarities between *Bsal* and *Bd* chytridiomycosis.

## 1. Introduction

Over the past five decades, chytridiomycosis has been responsible for the decline of >500 amphibian species, and the extinction of nearly 90 amphibian species, culminating in what has been described as the greatest documented loss of vertebrate biodiversity due to a pathogen (1). The culprit behind the majority of these drastic declines and extinctions has been the chytrid fungus *Batrachochytrium dendrobatidis* (*Bd*), which was first detected in the 1990's (1). In 2013, a novel chytrid fungus, *Batrachochytrium salamandrivorans* (*Bsal*), was discovered in fire salamanders (*Salamandra salamandra*), and is causing mass die-offs of several urodelean species in multiple European countries (2–4). This pathogen mainly infects urodeles; however, it has also been shown to infect and cause disease in anuran species (3, 5, 6).

*Bsal* presumably originated in Asia and is thought to have spread to Europe through international amphibian trade (7). This pathogen has not yet been detected in North America (8). However, studies report that likelihood of *Bsal* invasion is high due to suitable environmental conditions, a large number of susceptible host species, high prevalence of *Bsal* in heavily-traded amphibian species, and few trade regulations to reduce the risk of further global translocations (9–14). Despite recognition of the severe threat that *Bsal* poses to global amphibian biodiversity, we still know little about the mechanisms of its pathogenesis, limiting our ability to develop effective therapeutics to treat infected individuals.

While limited information has been reported on the pathophysiological effects of *Bsal*, studies of *Bd* have provided significant information on chytrid fungus pathophysiology. Studies of *Bd*, have shown that electrolyte imbalances occur in amphibians due to reduced osmoregulation through extensive skin pathology (15–19). These electrolyte losses have been shown to lead to cardiac arrest, which is likely the ultimate cause of mortality in *Bd*-infected individuals (18).

*Bd* and *Bsal* are closely related to each other yet differ in the type of skin lesions they induce (2, 20). Amphibians infected with *Bd* typically develop proliferative epidermal lesions that result in thickening of the skin, whereas amphibians infected with *Bsal* develop erosive to ulcerative, necrotizing lesions that create holes in the skin (2, 21). Although the type of skin injury is different for each pathogen, it is hypothesized that the mechanisms by which each pathogen causes morbidity and mortality have similarities because they are both impacting skin function. However, differences such as potential secondary bacterial infections and dermal gland invasion in *Bsal*-infected amphibians are also suspected (22, 23). Additionally, it has been suggested that anorexia may play a role in *Bsal* pathogenesis (24). Here, clinical presentation along with clinical and anatomic pathology changes are compared between diseased vs. non-diseased individuals by measuring blood cell counts, biochemical variables, and plasma protein levels in combination with histologic examination of all organs. By combining histological and molecular techniques along with clinical presentation, the goal was to gain new understanding of how *Bsal* chytridiomycosis causes morbidity and mortality in susceptible hosts.

## 2. Materials and methods

### 2.1. Ethics statement

Husbandry as well as euthanasia procedures followed recommendations provided by the American Veterinary Medical Association and the Association of Zoos and Aquariums. Additionally, they were approved by the University of Tennessee Institutional Animal Care and Use Committee (protocol #2623). *Taricha granulosa* that reached euthanasia endpoints were humanely euthanized *via* transdermal exposure to benzocaine hydrochloride.

In previous studies of fish, the use of benzocaine hydrochloride as an anesthetic agent was shown to have immunosuppressive effects (25). The current study controlled for this by including a control group not exposed to *Bsal* for comparison. Additionally, animals in the current study were exposed to a high dose of benzocaine hydrochloride for a short period of time as the end goal was euthanasia rather than anesthesia as it was in the aforementioned study in fish. Therefore, immunosuppressive effects are expected to be minimal and accounted for.

### 2.2. Animals

A total of 49 *Taricha granulosa* (rough-skinned newts) were wild-caught by California Department of Fish and Wildlife and shipped to the University of Tennessee. *Taricha granulosa* were used due to their known susceptibility to *Bsal* (7) and their relatively large size which allowed a sufficient amount of blood to be collected from each individual. Upon arrival to the laboratory, individuals were each given an individual ID (“TW1”-“TW49”). We confirmed that all *T. granulosa* were *Bd* negative by using quantitative polymerase chain reaction (qPCR) methods identical to those previously described (26). All animals were then heat-treated for 10 days at 30°C to clear any potential pre-existing *Bd* infections which may have not been detected by qPCR (22, 27). Co-infection with *Bd* can influence disease outcomes in *Bsal* chytridiomycosis (28); however, previous infection and clearance of *Bd* does not seem to have an effect on susceptibility to *Bsal* in other urodelan species (28). We again confirmed that all *T. granulosa* were *Bd* negative prior to the beginning of the trial by testing skin swabs using qPCR. The pre-trial swabs as well as all skin swabs performed throughout the study consisted of 10 swipes along the ventrum and 5 swipes on the bottom of each foot. After heat treatment, *T. granulosa* were acclimated for 2 weeks to a temperature of 14°C, which has been found to be the optimal temperature for *Bsal* growth and infection of urodelan species (2, 9). Animals were individually housed in an environmental growth chamber (Conviron, Winnipeg, Canada) and temperature was maintained at 14°C throughout the entire study. The environmental growth chamber also provided light/dark cycles reflecting the corresponding season associated with the environmental temperature (spring/fall = 14 h dark). Individual housing consisted of sterilized 710-cm^3^ containers that included a moist paper towel and PVC cover object. All containers, paper towels, and cover objects were replaced with sterile ones every 3 days. Diet consisted of medium-sized crickets (amount based on 4% of each animal's body weight measured at the beginning of study) given at each water change and food consumption was noted at the following water change.

### 2.3. Experimental design

**Figure 1:**
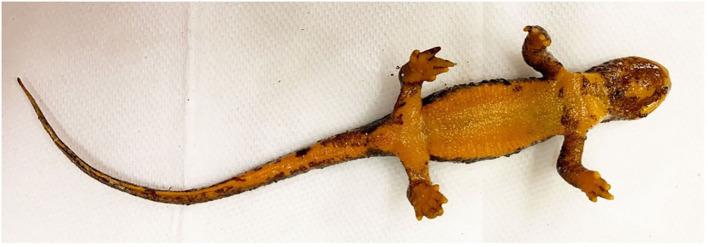
*Batrachochytrium salamandrivorans* exposed, clinically diseased *Taricha granulosa* with severe, multifocal skin sloughing especially prominent on the chin, neck, legs, feet, and tail (TW49).

**Figure 2:**
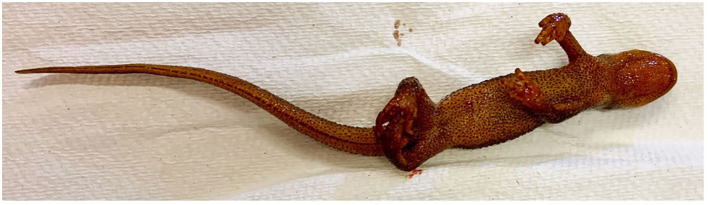
*Batrachochytrium salamandrivorans* exposed, clinically diseased *Taricha granulosa* with multifocal hemorrhage on the chin, legs, and feet (TW25).

**Figure 3:**
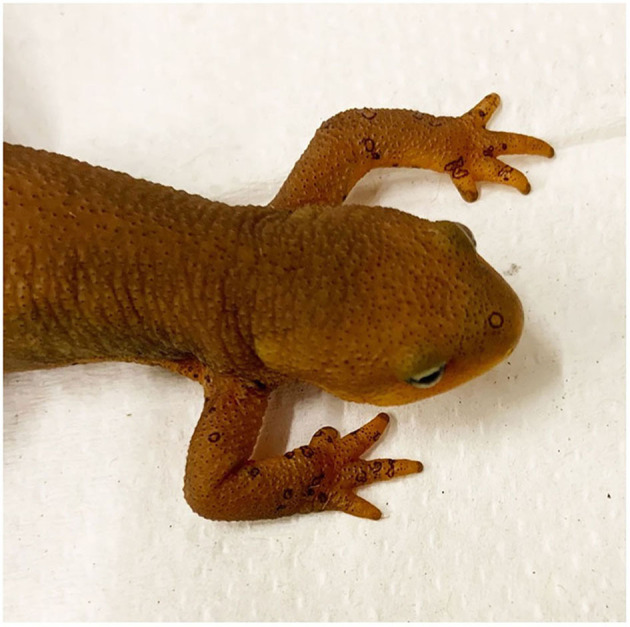
*Taricha granulosa* with multiple, characteristic erosive to ulcerative *Batrachochytrium salamandrivorans* induced skin lesions (TW14).

The 49 *T. granulosa* were randomly assigned to either a control (*n* = 8) or *Bsal*-exposed (*n* = 41) treatment group. Animals were monitored twice daily for signs of chytridiomycosis. Detailed notes regarding lesion coverage, skin sloughing, and degree of lethargy were taken and graded at each of these timepoints (skin sloughing and lesion coverage: 0 = none; 1 = < 25% body coverage, 2 = 25–50% body coverage, 3 = 51–75% body coverage, 4 = >75% body coverage; Lethargy grade: 0 = none; 1 = mild; 2 = moderate; 3 = severe). Examples of skin sloughing, hemorrhage, and multifocal epidermal ulcerations are provided in Figures 1–3, respectively. Those individuals that reached a pre-determined euthanasia endpoint (loss of righting ability) were humanely euthanized through transdermal administration of benzocaine. Others were euthanized at various stage of disease progression or at the end of the study (60 days). *Bsal*-exposed *T. granulosa* were classified as “clinically diseased” if they exhibited evidence of moderate to severe lethargy (i.e., loss of righting reflex) at the time of euthanasia. Control *T. granulosa* as well as *T. granulosa* which were *Bsal*-exposed, became infected and exhibited no to mild lethargy at the time of euthanasia (subclinically infected) were classified as “non-clinically diseased.” The final sample size used for analyses was 22 clinically diseased and 27 non-clinically diseased animals.

### 2.4. Experimental infection

The *Bsal* used in our experiments was isolated from a fire salamander (*Salamandra salamandra*; isolate AMFP13/1) in the Netherlands which was passed <25 times (2). Cultures were maintained at the University of Tennessee Center for Wildlife Health laboratory and zoospores were enumerated using previously described methods (24).

*Bsal*-exposed individuals were exposed to a dose of 1 × 10^7^ zoospores per 10 mL by being individually placed into a 100-mL plastic tube containing a mixture of *Bsal* zoospores and sterile water for 24 h in the environmental incubators. Control animals were placed into 100-mL plastic tubes containing 10-mL of sterile water under the same time and environmental conditions as the exposed individuals.

### 2.5. Sample collection

Following *Bsal* inoculation, animals were skin swabbed once every 3 days throughout the study for an estimate of infection load. Additionally, skin swabs were collected for all animals at the time of necropsy, and gross images were taken. As lighting in the room where the gross images were taken was dim, images were brightened using Adobe Photoshop (version 23.2.2) to aid in lesion detection. Immediately following euthanasia, tissues overlying the heart were carefully dissected using sterile instruments, and blood was collected directly from the heart using a sterile, heparinized capillary tube. Three to 5 microliters of blood were transferred onto a glass slide to make a blood smear using a gentle smear technique, five microliters of blood were pipetted into 995 microliters of filtered Natt-Herrick solution in a 1.5 milliliter sterile plastic conical tube, and the remaining blood sample was placed into a sterile 1.5 milliliter plastic conical tube. Blood samples in conical tubes were kept in a cooler on ice immediately after collection and transported to the University of Tennessee College of Veterinary Medicine (UTCVM) clinical pathology laboratory for processing. A sample of skin from the ventrum as well as a toe from each animal were collected and placed into separate 1.5 milliliter conical tubes. All skin swabs along with the skin and toe samples were placed immediately into a−80°C freezer. Carcasses were placed whole into 10% neutral buffered formalin.

### 2.6. Hematobiochemical analyses

All blood samples were processed in the University of Tennessee College of Veterinary Medicine (UTCVM) clinical pathology lab. Packed cell volume (PCV) was estimated using whole blood samples. White blood cell differentials were performed on blood smears stained with Wright's Giemsa stain, and white blood cell/red blood cell/platelet counts were performed on the blood in the Natt-Herrick solution using a hemocytometer as outlined in Nardini et al. (29). Cell counts were performed within 48 h of blood being mixed with Natt-Herrick solution. Whole blood was spun in an Eppendorf 5424R centrifuge at 2,170 G for 3 min to separate plasma. Plasma was analyzed using a Roche Cobas c501 analyzer assessing the sodium (Na), potassium (K), chloride (Cl), bicarbonate (CO_2_), total protein, magnesium (Mg), calcium (Ca), phosphorous, alkaline phosphatase (ALP), creatinine kinase (CK), cholesterol, blood urea nitrogen (BUN), glucose, lactate dehydrogenase (LDH), as well as the hemolytic, lipemic, and icteric indices. Remaining plasma was stored in a 4°C refrigerator until it was used for agarose gel electrophoresis (< 24 h after collection).

### 2.7. Plasma protein electrophoresis

Plasma samples were analyzed in accordance with instructions provided by the Helena QuickGel system with the use of Split Beta gels (Helena Laboratories, Inc., Beaumont, Texas 77707, USA). Samples were run in duplicate (average mean variance between duplicates = 0.28) and with a known human control sample. Gels were scanned and analyzed using software provided by Helena.

### 2.8. Histopathology

Carcasses were stored in 10% neutral buffered formalin for a minimum of 48 h prior to processing. Transverse sections through the entire body were taken and placed into tissue cassettes following the protocol listed in Table 1. Cassettes containing tissues were decalcified for 24 h using a formic acid solution. Tissues were then routinely processed and stained with hematoxylin and eosin (H&E). Histopathology consisted of examining all tissues for any abnormalities as well as performing *Bsal*-associated skin lesion counts on all sections. To standardize measures of lesion counts to counts per unit area of cross section, the perimeter of each section was determined using Excelis Accu-Scope software.

**Table 1:** Anatomic location associated with each of ten standardized sections examined histologically for lesion counts and grading in *Batrachochytrium salamandrivorans* infected *Taricha granulosa*.

**Section number**	**Anatomic location**
1	Cranial to eye
2	Caudal to eye
3	Cranial to thoracic limbs
4	Caudal to thoracic limbs
5	Left forelimb
6	Right hindlimb
7	Mid-body
8	Cranial to hindlimbs
9	Caudal to hindlimbs
10	Caudal 3 cm of the tail

Presence or absence of inflammation associated with skin lesions was documented for each exposed individual. Additionally, inflammation within each *Bsal*-associated lesion was estimated as mild (<30% of the lesion infiltrated by inflammatory cells), moderate (30–60% of the lesion infiltrated by inflammatory cells), or severe (>60% of the lesion infiltrated with inflammatory cells), and an overall severity grade was assigned to each individual based on which grade of inflammation occurred most frequently. Also, areas of inflammation associated with *Bsal* but not bacteria were counted for each individual.

### 2.9. Quantitative polymerase chain reaction

To detect *Bsal* and estimate loads, genomic DNA was extracted from each skin swab using the QIAamp 96 DNA QIAcube HT kit (Qiagen, Hilden, Germany) and qPCR was performed similar to previously described methods (30) using the Applied Biosystems Quantstudio 6 Flex qPCR instrument (Thermo Fisher Scientific Inc). All samples were run in duplicate and declared positive if both replicates reached cycle threshold prior to 50 amplification cycles (24).

### 2.10. Statistical analyses

#### 2.10.1. Biochemical, hematological, and plasma protein electrophoresis measurands

To determine how clinical disease status affected biochemical variables, we tested whether variables were significantly different between clinically diseased and non-clinically diseased *T. granulosa*. Biochemical variables included in the analysis were alkaline phosphatase (ALP), blood urea nitrogen (BUN), phosphorous, calcium, glucose, protein, cholesterol, sodium (Na), chloride (Cl), bicarbonate (CO_2_), anion gap, magnesium, creatinine kinase (CK), lactate dehydrogenase (LDH), and potassium (K). Hemolytic index (H) was also measured for each sample. Increased hemolysis in a sample can lead to artifactual elevations in CK, LDH, and K. Therefore, CK was independently regressed on H, LDH on H, and K on H and the residuals of these regressions were extracted. These residuals account for the variation in CK, LDH, and K not described by changes in hemolytic index (H). These new variables were used in place of original CK, LDH, and K variables in the analyses.

The data were scaled (e.g., zero mean and standard deviation of one) and K means clustering with the elbow method was used to determine the optimal number of clusters to use in order to group individuals based on similarity in their biochemical variables (31). Because a principal component analysis (PCA) of the biochemical variables showed that the majority of clinically diseased animals clustered together, we subsequently ran a MANOVA to test which biochemical variables significantly differed between clinically diseased and non-clinically diseased animals. Shapiro-Wilk normality tests were performed on all variables to assess for normality, and variables which failed this test were log transformed. If the variable still failed the normality test once log transformed, then the original value was used. We used Wilks' statistic to interpret MANOVA significance due to its robustness toward lack of normality and homogeneity of variance (32). Significant differences between clinically diseased and non-clinically diseased individuals were tested for each biochemical value. Thus, to account for type I error rate, false discovery rate was used to correct *P*-values from the MANOVA for specific biochemical variables (33). A family-wise type I error rate of α = 0.05 was used for all analyses. Arithmetic means for each variable were compared between clusters. All analyses were run in R (version 4.1.1).

To determine how *Bsal* qPCR load at the time of necropsy affected changes in biochemical variables, a permutational multivariate analysis of variance (PERMANOVA) was performed using the vegan package (34). Biochemical variables were used as the dependent variable and *Bsal* load at the time of necropsy as the independent variable. The log of *Bsal* load +1 was used as some of the load values were 0. This analysis was followed with correlation tests between each individual variable and *Bsal* load using Pearson's product moment correlation and corrected with false discovery rate.

A similar set of analyses as described above was performed for hematological and plasma protein electrophoresis variables. Hematological variables included in the analysis were absolute counts of segmented neutrophils (ACSegNeut), band neutrophils (ACBands), lymphocytes (ACLymphs), monocytes (ACMonos), eosinophils (ACEos), and basophils (ACBasos). For variables that included zeros, the log of the variable +1 was used. Plasma protein electrophoresis results included 8 fractions (Avr1–8). Increased hemolysis in a sample can lead to artifactual alterations in various plasma protein fractions in other species (35). Therefore, each fraction was independently regressed on the hemolytic index of the sample (H). For each regression in which H was determined to significantly explain variation in the plasma protein fraction (fractions 3, 5, and 8), the residuals of these regressions were extracted. These residuals account for the variation in each of these fractions not described by changes in hemolytic index (H). These new variables were used in place of the original protein fractions in the analyses.

#### 2.10.2. Food consumption

To determine how *Bsal* chytridiomycosis affected food consumption in *T. granulosa*, a binomial generalized linear mixed effects model was fit where the response variable was the combined vector of food consumed and food not consumed, and the predictor variable was the interaction term of time and treatment (clinically diseased vs. controls) with the random effect of individual ID. Only exposed, clinically diseased animals and controls were included in this analysis due to variations in survival time of exposed, non-clinically diseased individuals. The model was fit using the lme4 package in R (version 4.1.1). An alternative model was fit to the data where time and treatment were included as main effects instead of as an interaction term. The two models were compared using Akaike information criteria (AIC), where lower AIC indicates better predictive performance.

#### 2.10.3. Lesion count

To determine how *Bsal* qPCR load at the time of necropsy affected lesion count, a negative binomial generalized linear model was fit where the response variable was lesion count per cross section perimeter and the predictor variables were log(*Bsal* load), section number, and the random effect of individual ID. Section number included sections 1–10, corresponding to different regions of the animal's body (Table 1). A negative binomial distribution was used to describe lesion counts as initial analyses showed that lesion counts were over-dispersed relative to a Poisson distribution. In addition, lesion counts were obtained from histological cross sections with different perimeters, where a larger perimeter could lead to a higher number of counted lesions than a smaller perimeter even if the density of lesions per unit perimeter were the same. To account for this, an offset term of log(total perimeter) was included in the negative binomial regression (36), which allowed the model to include the mean density of lesions per cross section rather than the total abundance. The lesion count model was fit using the brms package in R (version 4.1.1) (37), with a weakly regularizing prior on the effect sizes for the predictor variables log(*Bsal* load) and section number. When fitting the models, all convergence was ensured by visually examining chains and checking that R_hat (the potential scale reduction factor) < 1.01 and effective sample size > 400 for all parameters (38). Five candidate models were fit to the data and the best predictive model was selected using Pareto smoothed importance sampling leave-one-out information (PSIS-LOO) (39). Models with lower PSIS-LOO indicate better predictive performance. Models including the following combinations of predictors were compared to the final model described above: 1) log(*Bsal* load), 2) log(*Bsal* load) and section number, 3) log(*Bsal* load) and the random effect of individual ID, and 4) the interaction term of log(*Bsal* load) and section number along with the random effect of individual ID.

## 3. Results

### 3.1. Biochemical variables

**Figure 4:**
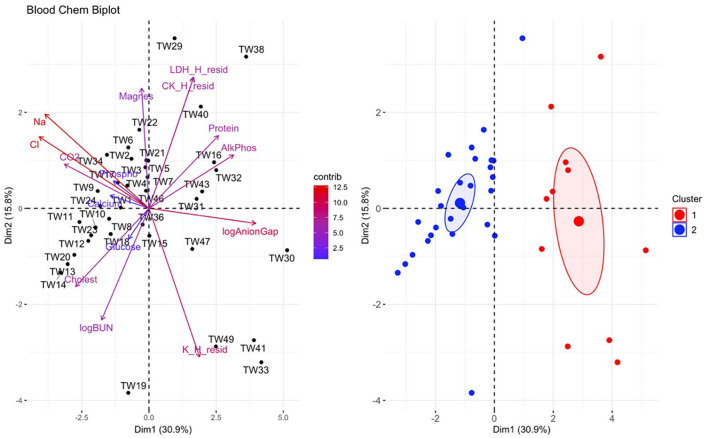
Principal component analysis (PCA) for biochemical data. In the biplot on the left, individuals in the same space as the arrowhead had higher values of the blood parameter, and individuals in the opposite direction had lower levels of the blood parameter. The length and color of each arrow represent the strength of the relationship. The plot on the right is showing how the individuals clustered together using K means clustering. The majority of *Taricha granulosa* in cluster 1 (red) were *Batrachochytrium salamandrivorans* (*Bsal*) infected, clinically diseased individuals, and the majority of *T. granulosa* in cluster 2 (blue) were controls or *Bsal*-infected, non-clinically diseased individuals. Na, Sodium; Cl, Chloride; cholest, Cholesterol; logBUN, Blood urea nitrogen; Phospho, Phosphorous; AlkPhos, Alkaline phosphatase; Magnes, Magnesium; K_H_resid, the residuals of potassium (K) regressed on hemolytic index (H); K_H_resid, the residuals of creatinine (CK) regressed on H; K_H_resid, the residuals of lactate dehydrogenase (LDH) regressed on H.

**Figure 5:**
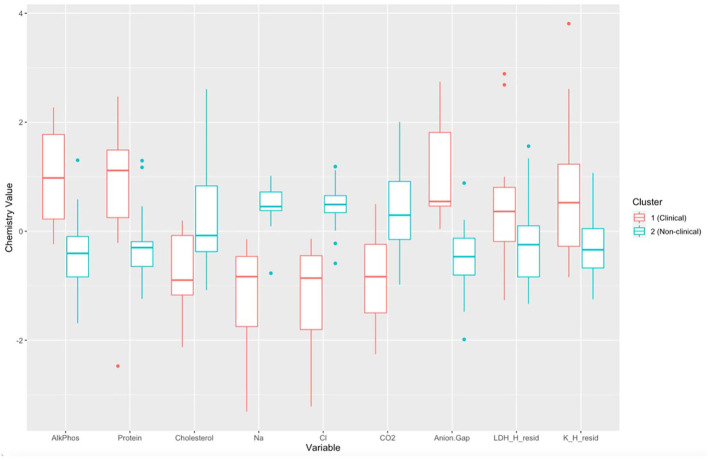
Boxplots comparing each biochemical variable for cluster 1 (*Batrachochytrium salamandrivorans* (*Bsal*) infected, clinically diseased *Taricha granulosa*) in red and cluster 2 (control and *Bsal*-infected, non-clinically diseased *T. granulosa*) in blue. Each box represents the interquartile range (IQR), the horizontal line within each box represents the median, vertical lines represent 1.5 ^*^ IQR, and dots represent outliers. AlkPhos, Alkaline phosphatase; Na, sodium; Cl, chloride; CO_2_, bicarbonate; LDH_H_resid, the residuals of lactate dehydrogenase (LDH) regressed on hemolytic index (H); K_H_resid, the residuals of potassium (K) regressed on H.

There was a significant difference in biochemical variables between the two clusters corresponding to clinically diseased and non-diseased individuals (PCA shown in Figure 4; MANOVA: Wilks' statistic = 0.13, *P* < 0.001). Na [*F*_(1, 36)_ = 53.8, *P* < 0.001], Cl [*F*_(1, 36)_ = 53, *P* < 0.001], CO2 [*F*_(1, 36)_ = 18, *P* < 0.001], and cholesterol [*F*_(1, 36)_ = 9.8, *P* = 0.004] were significantly decreased on average in clinically diseased animals compared to non-clinically diseased animals and K [*F*_(1, 36)_ = 11, *P* = 0.002], ALP [*F*_(1, 36)_ = 24.7, *P* < 0.001], anion gap [*F*_(1, 36)_ = 30, *P* < 0.001], total protein [*F*_(1, 36)_ = 10.4, *P* < 0.001], and LDH [*F*_(1, 36)_ = 4.9, *P* = 0.033] were significantly increased (Figure 5).

**Figure 6:**
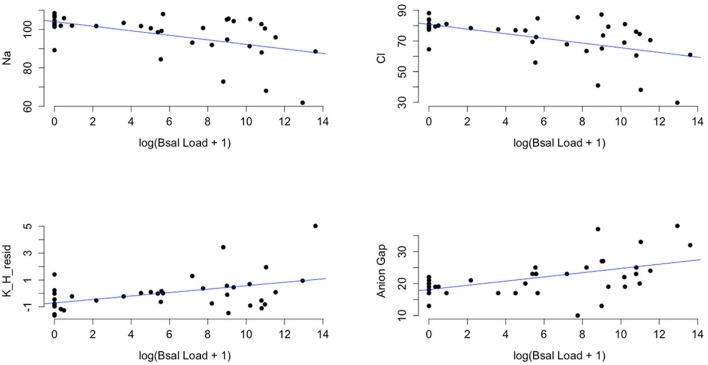
Plots of sodium (Na), chloride (Cl), the residuals of potassium (K) regressed on hemolytic index (H) (K_H_resid), and anion gap (y-axis) and the log of *Batrachochytrium salamandrivorans* qPCR load at time of necropsy + 1 (x-axis). Black points are observed data points and blue lines are best-fit regression lines for visual reference of the dominant trend in the data. Additional analysis showed that there was significant evidence for a non-linear quadratic effect of log(*Bsal* Load + 1) on biochemical variables (log(*Bsal* Load + 1)^2^ (effect from PERMANOVA: F = 6.98, *p* = 0.001).

There was a significant relationship between biochemical variables and *Bsal* load (PERMANOVA: f= 14.97, *p* = 0.01). The variables Na, Cl, K, and anion gap were significantly correlated with *Bsal* load at the time of necropsy (Na: *r* = −0.51, P = 0.001, 95% Confidence Interval (CI): −0.71– −0.22; Cl: *r* = −0.53, *P* < 0.001, 95% CI: −0.73 −0.26; K: *r* = 0.45, *P* = 0.005, 95% CI: 0.15–0.67; Anion gap: *r* = 0.45, *P* = 0.004, 95% CI: 0.15–0.67). As *Bsal* load at necropsy increased, K and anion gap increased, and Na and Cl decreased (Figure 6).

### 3.2. Hematological vales

**Figure 7:**
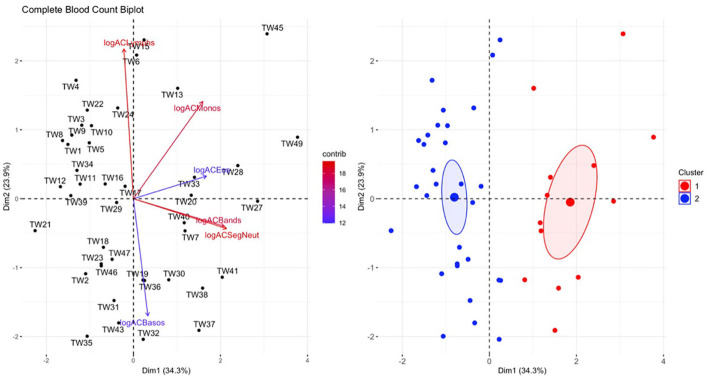
Principal component analysis (PCA) for the complete blood count (CBC) data. In the plot on the left, individuals in the same space as the arrowhead had higher values of the blood parameter, and individuals in the opposite direction had lower levels of the blood parameter. The length and color of each arrow represent the strength of the relationship. The plot on the right is showing how the individuals clustered together using K means clustering. The majority of *Taricha granulosa* in cluster 1 (red) were *Batrachochytrium salamandrivorans* (*Bsal*) infected, clinically diseased individuals, and the majority of *T. granulosa* in cluster 2 (blue) were controls or *Bsal*-infected, non-clinically diseased individuals. logACLymphs, Absolute count of lymphocytes; logACMonos, absolute count of monocytes; logACEos, absolute count of eosinophils; logACBands, absolute count of band neutrophils; logACSegNeut, absolute count of segmented neutrophils; logACBasos, absolute count of basophils.

**Figure 8:**
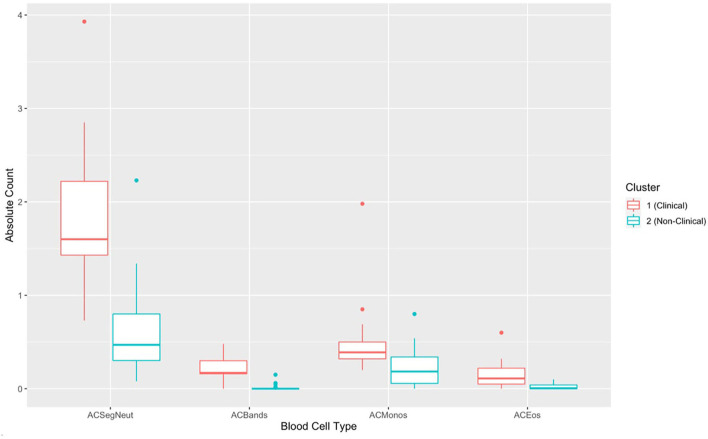
Boxplots comparing each complete blood count variable for cluster 1 (*Batrachochytrium salamandrivorans* (*Bsal*) infected, clinically diseased *Taricha granulosa*) in red and cluster 2 (controls and *Bsal*-infected, non-clinically diseased *T. granulosa*) in blue. Each box represents the interquartile range (IQR), the horizontal line within each box represents the median, vertical lines represent the 1.5 ^*^ IQR, and dots represent outliers. ACSegNeuts, Absolute count of segmented neutrophils; ACBands, absolute count of band neutrophils; ACMonos, absolute count of monocytes; ACEos, absolute count of eosinophils.

There was a significant difference in hematological variables between clinically diseased and non-clinically diseased clusters (PCA shown in Figure 7; MANOVA: Wilks' statistic: 0.22, *P* = < 0.001). Segmented neutrophils [*F*_(1, 41)_ = 27.8, *P* < 0.001], band neutrophils [*F*_(1, 41)_ = 54.1, *P* < 0.001], monocytes [*F*_(1, 41)_ = 12.64, *P* < 0.001], and eosinophils [*F*_(1, 41)_ = 18, *P* < 0.001] were significantly increased in the majority of clinically diseased animals compared to non-clinically diseased animals (Figure 8).

**Figure 9:**
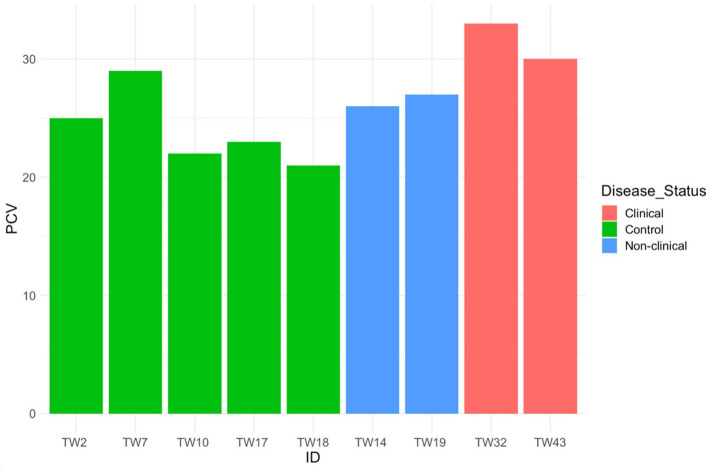
Packed cell volume for five control, two *Batrachochytrium salamandrivorans*-exposed/non-clinically diseased, and two *Batrachochtyrium salamandrivorans*- exposed/clinically diseased *Taricha granulosa*.

Mild toxic change was identified in neutrophils of 3 exposed, clinically diseased along with 1 control *T. granulosa*. Döhle bodies (1+) were identified in 1 exposed, clinically diseased and 1 control *T. granulosa*. While trends in the means of PCV showed differences between controls, exposed/non-clinically diseased and exposed/clinically diseased *T. granulosa* (Figure 9), only two clinically diseased individuals had PCV measurements and thus a formal statistical analysis was not performed. Small sample size for PCV analysis was due to limitations with blood volume collected.

### 3.3. Plasma protein electrophoresis

**Figure 10:**
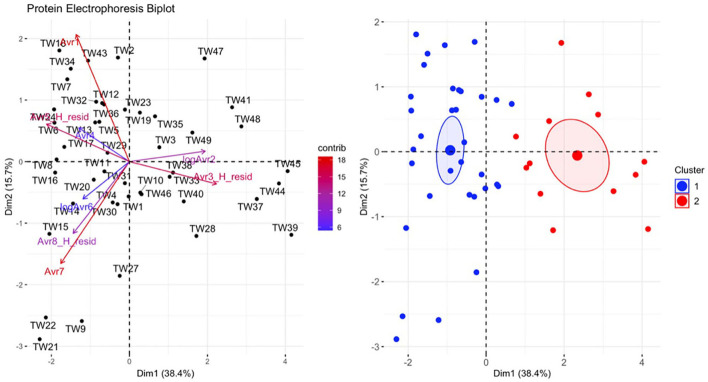
Principal component analysis (PCA) for the plasma protein electrophoresis data. In the plot on the left, individuals in the same space as the arrowhead had higher values of the blood parameter, and individuals in the opposite direction had lower levels of the blood parameter. The length and color of each arrow represent the strength of the relationship. The plot on the right is showing how the individuals clustered together using K means clustering. The majority of *Taricha granulosa* in cluster 1 (blue) were controls or *Batrachochytrium salamandrivorans (Bsal)* infected, non-clinically diseased individuals, and the majority of *T. granulosa* in cluster 2 (red) were *Bsal*-infected, clinically diseased individuals.

**Figure 11:**
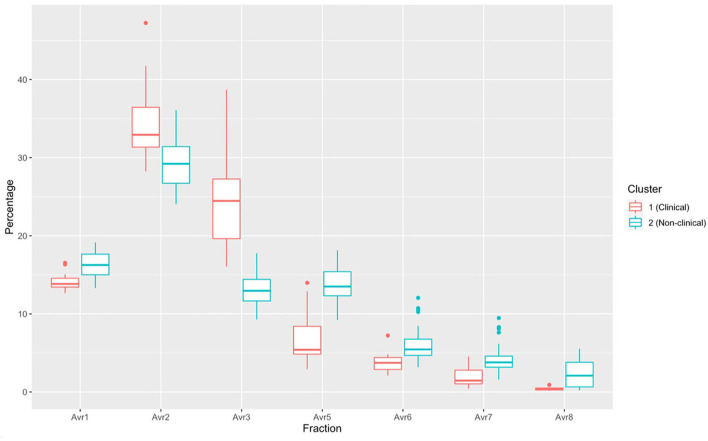
Boxplots comparing each protein electrophoresis fraction for cluster 1 (*Batrachochytrium salamandrivorans* (*Bsal*) infected, clinically diseased *Taricha granulosa*) in red and cluster 2 (controls and *Bsal*-infected, non-clinically diseased *T. granulosa*) in blue. Each box represents the interquartile range (IQR), the horizontal line within each box represents the median, vertical lines represent 1.5 ^*^ IQR, and dots represent outliers.

**Figure 12:**
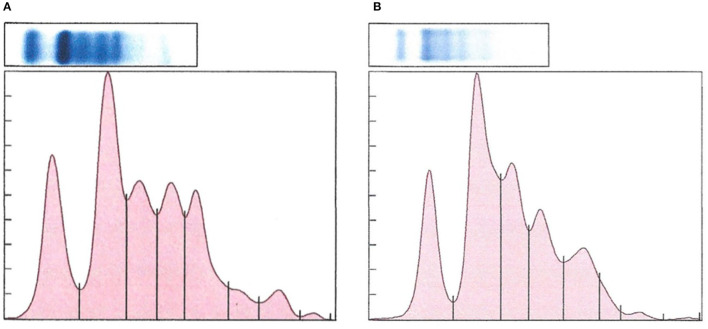
Representative protein electrophoretograms from a control **(A)** and a *Batrachochytrium salamandrivorans* infected, clinically diseased **(B)** *Taricha granulosa*.

There was a significant difference in plasma protein electrophoresis variables between the diseased and non-diseased clusters (PCA shown in Figure 10; MANOVA: Wilks' statistic: 0.17, *P* < 0.001). Protein fractions two [*F*_(1, 44)_ = 20.7, *P* < 0.001] and three [*F*_(1, 44)_ = 40.7, *P* < 0.001] were, on average, significantly increased in clinically diseased animals compared to non-clinically diseased animals, and fractions one [*F*_(1, 44)_ =14.2, *P* < 0.001], five [*F*_(1, 44)_ = 34.5, *P* < 0.001], six [*F*_(1, 44)_ = 18.3, *P* < 0.001], seven [*F*_(1, 44)_ = 15.5, *P* < 0.001], and eight [*F*_(1, 44)_ = 7.5, *P* = 9.96e-03] were decreased (Figure 11). Figure 12 shows an example electrophoretogram from a clinically diseased and a control *T. granulosa*.

### 3.4. Food consumption

**Figure 13:**
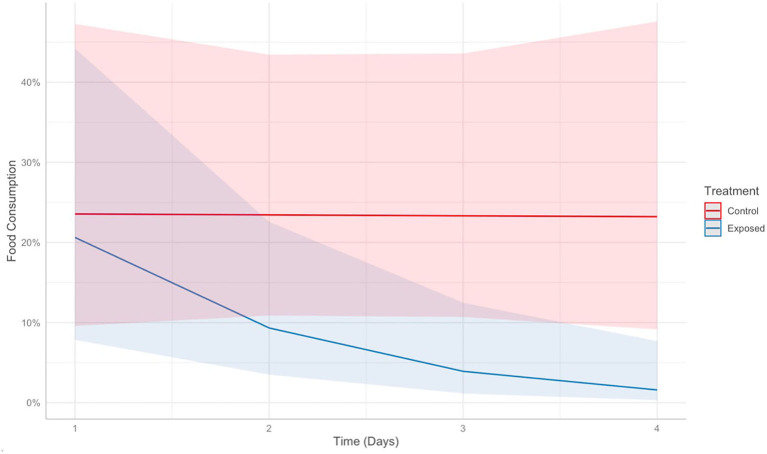
Predicts food consumption over time (days) post-exposure in control (red) vs. *Batrachochytrium salamandrivorans* exposed (blue) *Taricha granulosa*. Colored lines represent mean probabilities and shaded regions are 95% confidence intervals around the mean probabilities.

A strong interaction was identified between time and *Bsal* exposure on food consumption in control vs. clinically diseased *T. granulosa* (Effect size: −0.92, *Z* = −2.59, *P* = 0.009). The food consumption of control individuals remained consistent over time, while food consumption of *Bsal*-exposed individuals decreased (Figure 13). This was supported by model comparison – the AIC of the model including time^*^treatment was 5.44 units lower than the model including time + treatment.

### 3.5. Anatomic pathology

#### 3.5.1. Lesion counts

**Figure 14:**
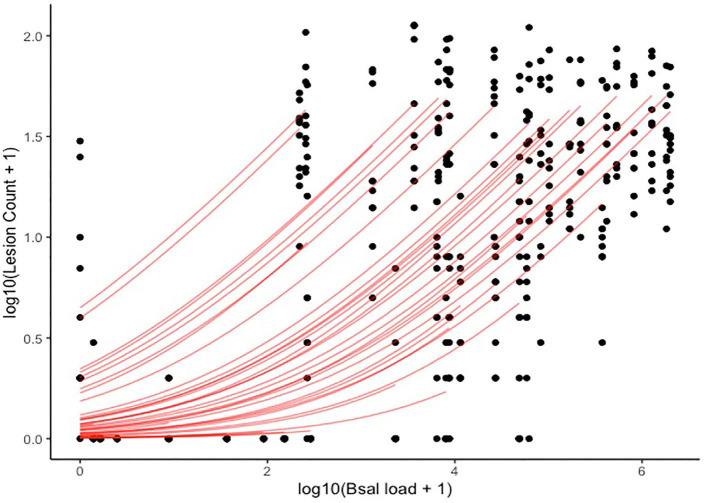
Plot of log10(Lesion count + 1) (y-axis) and log10(*Bsal* load + 1) (x-axis). Black points represent histologic lesion count at each of 10 standardized anatomical sections examined for each *Batrachochytrium salamandrivorans* (*Bsal*) infected *Taricha granulosa*. Red lines represent individual level relationships as predicted by our statistical model. As each individual only had one *Bsal* qPCR load (at necropsy), but multiple lesion counts due to the multiple sections, a unique slope cannot be inferred for the relationship between *Bsal* load and lesion count for each individual.

**Figure 15:**
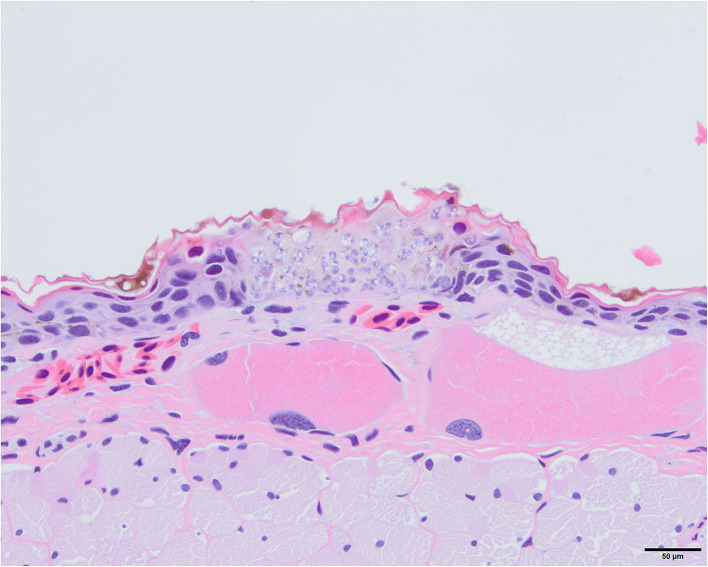
Photomicrograph stained with hematoxylin and eosin (H&E) of a skin lesion caused by *Batrachochytrium salamandrivorans* (*Bsal*) chytridiomycosis. There is full thickness invasion of the epidermis by *Bsal* thalli along with associated epithelial damage which is characteristic of skin lesions caused by this pathogen.

A significant positive relationship was identified between lesion count and *Bsal* load at necropsy (Figure 14), with the best model (i.e., the model with the lowest PSIS-LOO) including log_10_(*Bsal* load), section number, and the random effect of individual ID as predictors. Other candidate models included the following predictors, with the difference in PSIS-LOO between each candidate model and the best model listed: (1) log_10_(*Bsal* load)^*^section number + random effect of individual ID = 1.1, (2) log_10_(*Bsal* load) + random effect of individual ID = 18.5, (3) log(*Bsal* load) = 349.4, (4) log_10_(*Bsal* load) and section number = 354.2. The best model predicted that a one unit increase in log_10_(*Bsal* load) led to a 2.84X increase in lesion count (95% CI: 2.01–4.45). A histologic example of a skin lesion caused by *Bsal* chytridiomycosis is provided in Figure 15.

#### 3.5.2. Inflammation associated with skin lesions

Inflammation was associated with *Bsal* skin lesions in 46% of *Bsal*-exposed individuals. Inflammation was frequently associated with *Bsal* lesions which also had secondary bacterial invasion. All inflammatory lesions associated with *Bsal* infection consisted of mononuclear as well as granulocytic inflammatory cells.

#### 3.5.3. Dermal glands and internal organs

**Figure 16:**
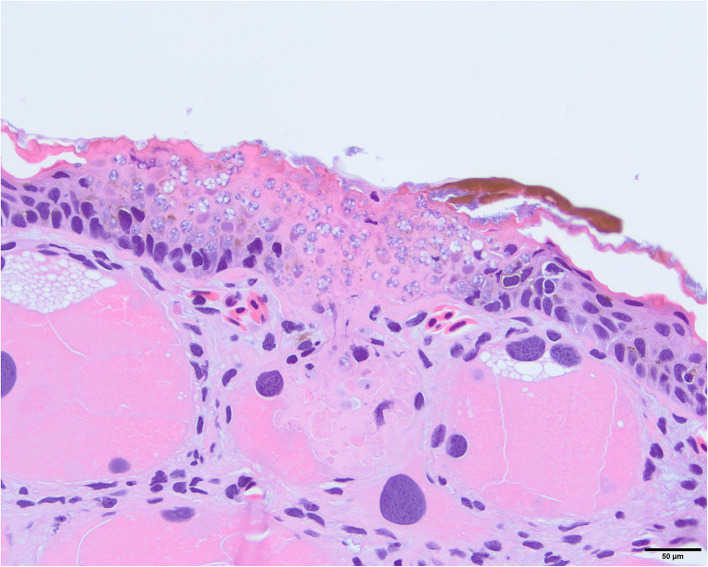
Photomicrograph stained with hematoxylin and eosin (H&E) showing invasion of *Batrachochytrium salamandrivorans* (*Bsal*) thalli into a dermal granular gland. There is full thickness invasion of the overlying epidermis by *Bsal* thalli along with associated epithelial damage.

Varying degrees of dermal gland invasion by *Bsal* organisms (from 1 to 13 glands total) were identified histologically in 75% of clinically diseased *T. granulosa*. Dermal gland invasion was only documented in one individual that was not classified as clinically diseased; however, did exhibit mild skin sloughing. This individual had *Bsal* organisms present in 4 dermal glands. All areas of dermal gland invasion had *Bsal* organisms and associated cellular damage in the overlying dermis and epidermis which was variably eroded to ulcerated (Figure 16). No lesions related to *Bsal* chytridiomycosis were identified in internal organs.

## 4. Discussion

The objective of this study was to elucidate the pathogenesis of *Bsal* chytridiomycosis through comparison of clinical presentation as well as clinical and anatomic pathology changes in diseased vs. non-clinically diseased individuals. Electrolyte imbalances and dehydration were identified as likely playing a key role in *Bsal* chytridiomycosis developing. Evidence of a systemic inflammatory response was also identified which may be in response to the *Bsal* organism itself, or due to secondary bacterial infection of *Bsal*-induced skin lesions.

Clinically diseased *T. granulosa* had significantly decreased blood Na and Cl levels and increased blood K levels compared to control and non-clinically diseased *T. granulosa*. Changes in Na and Cl are similar to those reported in *Bd* chytridiomycosis (15, 18, 19). However, in contrast to findings in *Bsal* chytridiomycosis, previous studies have reported decreased K levels in clinically diseased individuals with *Bd* chytridiomycosis compared to control and non-clinically diseased individuals (15, 18, 19).

The difference in distribution of lesions within the epidermal layers between *Bd* and *Bsal*, along with primarily immature sporangia infecting this critical layer in *Bd* infections, may contribute to the differences noted in blood K concentrations between the two pathogens. *Bd* thalli infect epidermal cells of the stratum corneum and stratum granulosum, with mature sporangia infecting cells of the stratum corneum and immature sporangia infecting the stratum granulosum (40). The stratum granulosum is the layer of the epidermis most prominently involved in the active transport of electrolytes and regulates electrolyte balance and osmoregulation in amphibians (16, 41). In contrast, *Bsal* thalli including mature forms frequently extend into deeper layers of the epidermis and in severe lesions, the epithelial layer is absent due to ulceration (42). Therefore, the extent of damage to this layer of the epithelium is likely more extensive in *Bsal*-infected individuals, which may lead to the differences in K concentrations seen between these two pathogens.

Another explanation for observed differences in K between hosts with *Bd* and *Bsal* chytridiomycosis might be related to the model species–most studies investigating electrolyte imbalances with *Bd* chytridiomycosis have used anurans (1). However, this study used a urodelan as the model, since urodelans have been shown to be more susceptible to *Bsal* infection (7). It has been shown that ion transport characteristics, particularly involving K, can vary between species and also with environmental conditions in amphibians (43). Additionally, the vast majority of what we know about electrolyte transport within amphibian epidermal cells is based on studies in anurans. Therefore, it is possible that there are intrinsic differences in epidermal electrolyte transport between frogs and salamanders, contributing to differences in K concentrations.

Another important finding in clinically diseased *T. granulosa* was increased total protein in biochemical analyses along with increased fractions 2 and 3 and secondarily decreased fractions 1, 5, 6, 7, and 8 in plasma protein electrophoretograms. Fraction 2 is believed to represent albumin based on an extrapolation of what is known in mammalian and more well-studied non-mammalian species. Increase in this fraction is most commonly associated with dehydration, especially in conjunction with increased total protein (44). Previous studies with *Bd* have not shown changes in total protein levels in clinically diseased individuals (15, 18, 19). However, some studies have shown evidence that dehydration may play a role in *Bd* chytridiomycosis pathogenesis. Young et al. (17) found that fluid therapy is useful to incorporate in successful treatment regimens for *Bd* chytridiomycosis (17). Additionally, another study provided evidence that clinically diseased *Litoria ranifomis* experienced slower rates of rehydration than non-clinically diseased individuals after *Bd* exposure, which may provide evidence that aquaporin pumps within the skin are impaired in diseased individuals (45).

Plasma protein fraction 3 in *T. granulosa* corresponds with the alpha-1 globulin fraction in other species. An increase in alpha-1 globulins is associated with either acute inflammation or parasitism in avian species (44). As no parasites were identified in *T. granulosa* in this study, acute inflammation is the most likely cause of this trend seen in clinically diseased individuals. This premise is also supported by the complete blood count results. Clinically diseased *T. granulosa* had increased segmented neutrophils, band neutrophils, monocytes, and eosinophils compared to control and non-clinically diseased *T. granulosa*. These increases likely represent an acute inflammatory response; however, it is uncertain whether this response is due to the *Bsal* infection itself or to a secondary bacterial infection (46).

One particularly interesting feature of the alterations identified in complete blood counts of clinically diseased *T. granulosa* is that they exhibited a left shift with increased numbers of band (immature) neutrophils in circulation. The presence of a left shift is most commonly associated with inflammation and occurs when inflammatory cytokines stimulate neutrophil production and release of mature and immature neutrophils from hematopoietic tissues (47, 48). The increase in band neutrophils in addition to increase in segmented (mature) neutrophils and other complete blood cell count findings further support this as an inflammatory response.

Another indication of an inflammatory response is that mild toxic change was identified in neutrophils of three clinically diseased *T. granulosa*, and a small number of Döhle bodies were identified in another clinically diseased *T. granulosa*. Toxic change in non-mammalian vertebrate species is similar to that in mammalian species and is caused by rapid release of neutrophils from hematopoeitic tissues into circulation which shortens their maturation time (48). Toxic change and a left shift often occur simultaneously as signs of an inflammatory response (48). Döhle bodies can also be an indication of accelerated maturation. All the clinically diseased *T. granulosa* who had a mild toxic change or the presence of Döhle bodies also had a left shift. Mild toxic change and a small number of Döhle bodies were also identified in two separate control individuals. Therefore, the significance of these findings is uncertain. Phagocytosed material or artifactual granulation due to suboptimal sample handling can be mistaken for Döhle bodies. Additionally, suboptimal staining or sample handling such as prolonged or improper storage can lead to other characteristics of toxic change although there was no evidence of these occurrences in our samples. Overall, further investigation of these changes is warranted to determine their significance.

To our knowledge, this is the first study investigating changes in complete blood count variables associated with *Bsal* chytridiomycosis. Studies with *Bd* chytridiomycosis have shown mixed results regarding alterations in circulating white blood cells associated with infection. Davis et al. (49) and Peterson et al. (50) showed neutrophilia and eosinopenia in *Bd*-infected animals (49, 50). Peterson et al. (50) also showed lymphocytopenia (50). Woodhams et al. (51) showed neutropenia and eosinopenia with basophilia in response to *Bd* infection (51). Overall, none of these previous studies with *Bd* have identified a similar inflammatory pattern to that seen in this study.

Previous studies of *Bd* and *Bsal* chytridiomycosis have shown no to minimal inflammation associated with skin lesions histologically. Overall, inflammation is uncommon, and when seen, is often associated with *Bd*-tolerant species or with secondary bacterial or fungal infections (42). In this study, inflammation was identified histologically within *Bsal*-associated skin lesions in 46% of *Bsal*-exposed *T. granulosa*. The vast majority of inflamed lesions also contained bacteria. Inflammation was frequently more severe in individuals that developed lesions, sloughed their skin and re-developed lesions without developing any evidence of lethargy or hemorrhage (likely indicating higher tolerance to infection), whereas inflammation was mild to absent in clinically diseased individuals. As the presence of inflammation may be associated with reduced *Bsal*-induced disease, this result suggests that stronger inflammatory processes may be observed in individuals with greater tolerance to infection (46, 52).

The majority of clinically diseased animals either died or were euthanized within 13 days post-exposure, and these individuals either died or were euthanized within 24 h of the onset of clinical disease. Therefore, it is possible that in these individuals, the immune system did not have time to appropriately respond to infection. Previous studies have provided evidence that *Bd* causes dysregulation of the local immune response within the skin, and that *Bsal* also possesses similar properties (53, 54). It is possible that as the local immune response was inadequate to fight off the infection, the systemic immune response was initiated; however, the immune cells did not have time to reach their destination prior to mortality of the individual. This hypothesis is supported by the increase in circulating leukocytes along with a left shift identified in clinically diseased animals. Thus, resistance to *Bsal* chytridiomycosis might be related to effective and rapid innate immune responses or to other factors (e.g., anti-microbial peptide production) that limit infection load or bacterial infiltration into the body.

Regarding additional biochemical variables, we also observed an increase in anion gap, LDH, and ALP, along with a decrease in bicarbonate and cholesterol in clinically diseased *T. granulosa*. Changes in anion gap and bicarbonate cannot be fully evaluated without the incorporation of blood gas analysis which was not performed in this study. This is because blood gas analysis evaluates the respiratory component of the acid-base status as well as the pH of the animal. Therefore, only speculations can be made based on the information obtained.

Potential reasons for increased anion gap in clinically diseased *T. granulosa* include increased albumin due to dehydration or increased accumulation of lactic acid due to cellular damage (e.g., skin damage). Additionally, if respiration is compromised due to epidermal damage, increased carbon dioxide within the body could contribute to a respiratory acidosis (47). Reasons for decreased bicarbonate could include either an increase in carbon dioxide secondary to respiratory compromise, or metabolic acidosis (47). These results suggest that cutaneous respiration could be compromised in salamanders with *Bsal* chytridiomycosis, which may have minimal effects on species with lungs (as in this study) but could be a substantial contributor to pathogenesis in lungless species. Metabolic acidosis could be due to an increase in acids such as lactic acid or a loss of bicarbonate through the gastrointestinal tract or kidneys. As no abnormalities were identified in the gastrointestinal or renal tract, this is unlikely in this case. Overall, future studies should incorporate blood gas analysis into diagnostic investigations of *Bsal* chytridiomycosis to further investigate the significance of these findings.

Lactate dehydrogenase can be increased due to tissue damage (e.g., skin damage) (47). It can also be increased in cases of liver or muscle injury and in some cases of neoplasia (47). No evidence of liver or muscle injury was identified; however, it is possible that hypoperfusion to these organs secondary to dehydration was occurring and leading to damage which was not yet visible histologically. Additionally, alkaline phosphatase can be increased in liver disease, particularly cholestasis (55). It can also be increased in association with increases in corticosteroids due to stress in some species such as dogs (55). Liver disease which was not evident histologically or increased corticosteroids secondary to the stress of being diseased could have contributed to this increase in clinically diseased *T. granulosa*. A previous study showed an increase in corticosteroid levels in salamanders exposed to *Bsal* (56). Therefore, measurement of corticosteroid levels would be useful in future investigations to help understand the contribution that stress could have on biochemical as well as complete blood count variables in *Bsal-*infected individuals.

Clinically diseased *T. granulosa* had decreased cholesterol compared to control and non-clinically diseased *T. granulosa*. This is suspected to be secondary to decreased food intake, as exposed, clinically diseased individuals consumed less food over time than control individuals in this study. This is an important finding, because malnutrition due to decreased food consumption in *Bsal*-infected individuals could contribute to morbidity and mortality (24).

The strong, positive relationship between *Bsal* qPCR load at necropsy and histologic lesion count shows that qPCR results are a good indicator of disease severity in *T. granulosa*. This is important as qPCR can be used as an antemortem diagnostic test, which is non-invasive because samples are easily collected through swabbing the skin. It has been shown previously that enumeration of grossly visible skin lesions associated with *Bsal* infection is only weakly, positively correlated with *Bsal* qPCR load at the time of necropsy in *Taricha granulosa* as well as three other salamander species including *Notophthalmus viridescens, Notophthalmus meridionalis*, and *Notophthalmus perstriatus* (52). As tolerance and resistance to infection can vary among species, it is important for additional studies to incorporate histologic lesion counts and test whether a consistent relationship exists with *Bsal* infection loads across multiple species (57).

Another important result related to *Bsal* qPCR load at necropsy is that as load increased, Na and Cl decreased, and K and anion gap increased. This further confirms the proposed pathogenesis of *Bsal* chytridiomycosis involving increased pathogen load leading to increased lesion count. We propose that increased epidermal damage due to increased lesion count, then leads to alterations in blood chemistry and ultimately clinical disease through a similar pathogenesis as to what has been described in reports of *Bd* chytridiomycosis (15–19).

As has been reported in previous chytridiomycosis studies, no internal lesions related to *Bsal* infection were identified (21, 58). This further supports the hypothesis that skin lesions are the primary cause of morbidity and mortality in *Bsal* chytridiomycosis. Histologic lesions associated with electrolyte imbalances and dehydration are often not present in internal organs especially in acute cases of morbidity and mortality as was seen in this study. Indeed, the lack of internal lesions provides important insight into *Bsal* pathogenesis.

Another potential contributor to *Bsal* disease pathogenesis is damage to the dermal glands. Seventy-five percent of clinically diseased *T. granulosa* had some degree of dermal gland invasion, whereas among non-clinically diseased *T. granulosa*, only one individual had evidence of dermal gland invasion. Dermal glands serve important functions such as maintaining moisture level of the skin, secreting antimicrobial peptides to inhibit infections, and producing toxins to deter predators (59). It is therefore possible that damage to these glands may lead to dehydration through decreased production of moisture as well as decreased ability to reduce water loss into the environment through evaporation. Previous studies have shown infection of dermal glands can occur in eastern newts (*Notophthalmus viridescens*) with *Bsal* chytridiomycosis, and it is suspected that this may play a role in disease progression (23). Investigation of compromise to dermal gland function with *Bsal* infection is warranted in future studies to better understand the role this may play in disease pathogenesis.

In summary, our study provides insight into the pathogenesis of *Bsal* chytridiomycosis through utilization of multiple diagnostic techniques. Based on these findings, there is evidence that *Bsal*-induced skin lesions lead to electrolyte imbalance and dehydration. It is possible that secondary bacterial infections and damage to dermal glands play a role in pathogenesis as well. Similar investigations involving additional species with various levels of *Bsal* infection tolerance are warranted to better understand the host response to disease. Additionally, studies utilizing additional diagnostic techniques, such as blood gas analysis and corticosteroid measurement, would be useful to determine what role respiratory compromise and stress response play in disease progression.

## Data availability statement

The raw data supporting the conclusions of this article will be made available by the authors, without undue reservation.

## Ethics statement

The animal study was reviewed and approved by University of Tennessee Institutional Animal Care and Use Committee.

## Author contributions

WS, MW, CC, EC, MG, and DM conceived the ideas and designed methodology. WS and EC collected the data. WS, MW, CC, and EC analyzed the data. WS led the writing of the manuscript. MG and DM secured funding for this project. All authors contributed critically to the drafts and gave final approval for publication.
