# Corrigendum: Electrolyte imbalances and dehydration play a key role in *Batrachochytrium salamandrivorans* chytridiomycosis

**DOI:** 10.3389/fvets.2023.1164389

**Published:** 2023-03-07

**Authors:** Wesley C. Sheley, Matthew J. Gray, Mark Q. Wilber, Carolyn Cray, E. Davis Carter, Debra L. Miller

**Affiliations:** ^1^Department of Biomedical and Diagnostic Sciences, College of Veterinary Medicine, University of Tennessee, Knoxville, Knoxville, TN, United States; ^2^Center for Wildlife Health, University of Tennessee Institute of Agriculture, Knoxville, TN, United States; ^3^Division of Comparative Pathology, University of Miami Miller School of Medicine, Miami, FL, United States

**Keywords:** amphibian, chytrid, clinical pathology, disease, histopathology, newt

A correction has been made to **Abstract**. This sentence previously stated:

“*Taricha granulosa* were exposed to a 1 x 107 per 10 mL dose”

The corrected sentence appears below:

“*Taricha granulosa* were exposed to a 1 x 10^7^ per 10 mL dose”

The authors apologize for this error and state that this does not change the scientific conclusions of the article in any way. The original article has been updated.

